# Automated cardiac motion estimation from 3D Cine DENSE MRI

**DOI:** 10.1186/1532-429X-14-S1-W16

**Published:** 2012-02-01

**Authors:** Andrew D Gilliam, Xiaodong Zhong, Frederick H Epstein

**Affiliations:** 1 A.D. Gilliam Consulting, Providence, RI, USA; 2 MR R&D Collaborations, Siemens Healthcare, Atlanta, GA, USA; 3 Biomedical Engineering, University of Virginia, Charlottesville, VA, USA; 4 Radiology, University of Virginia, Charlottesville, VA, USA

## Summary

The first fully automated solution for the estimation of myocardial mechanics from 3D cine DENSE MR data was developed. Results from the automated method agreed closely with a conventional analysis which requires manual segmentation of cardiac anatomy.

## Background

3D cine displacement encoding with stimulated echoes (DENSE) directly encodes tissue displacement into MR phase data, providing a comprehensive 3D view of cardiac motion and strain. Unfortunately, 3D cine DENSE motion analysis presently requires manually delineated anatomy. An automated analysis would reduce inter-observer variability, improve measurement throughput, and simplify data interpretation. This research develops the first fully automated solution for the estimation of myocardial mechanics from 3D cine DENSE data.

## Methods

3D cine DENSE CMR data were acquired in five healthy volunteers using a 1.5T MR system (Avanto, Siemens). All imaging was performed in accordance with protocols approved by our Institutional Review Board and with informed consent.

DENSE observes tissue displacement at fixed spatial locations through which the underlying tissue moves. Each DENSE phase observation is proportional to a displacement indicating the initial location of underlying tissue at end-diastole. Large phase values due to large displacements are wrapped to the intrinsic phase range of [-π,π]. Regions devoid of tissue contain unpredictable phase information.

The automated algorithm estimated 3D material point trajectories from the wrapped and un-segmented DENSE observations without the need for user interaction. Candidate material targets were initialized at evenly spaced locations within the 3D field-of-view without knowledge of tissue presence. For each frame, the algorithm predicted target positions guided by prior target motion, unwrapped DENSE observations consistent with neighboring predictions, and estimated true target positions from unwrapped observations via 3D compact support radial basis function interpolation. Candidate targets far from valid unwrapped DENSE observations or in regions of low DENSE magnitude are likely devoid of tissue and were discarded. This process was repeated for each frame of the 3D cine DENSE dataset, and the entire procedure was repeated to further refine target motion.

## Results

**Figure 1:**
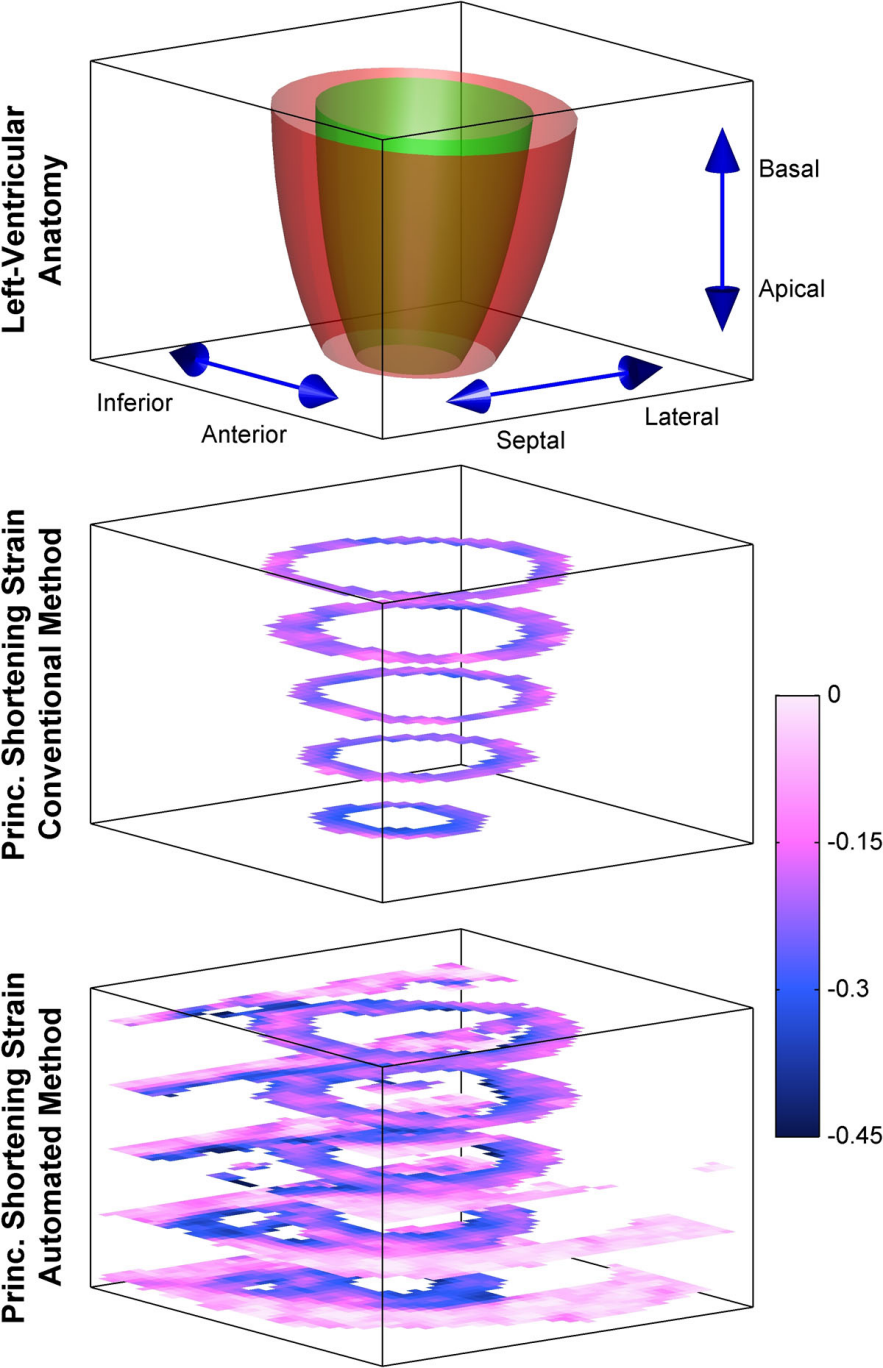
End-systolic principle shortening strain patterns (3rd principle strain) in a normal heart derived from conventional and automated 3D cine DENSE analyses.

**Figure 2:**
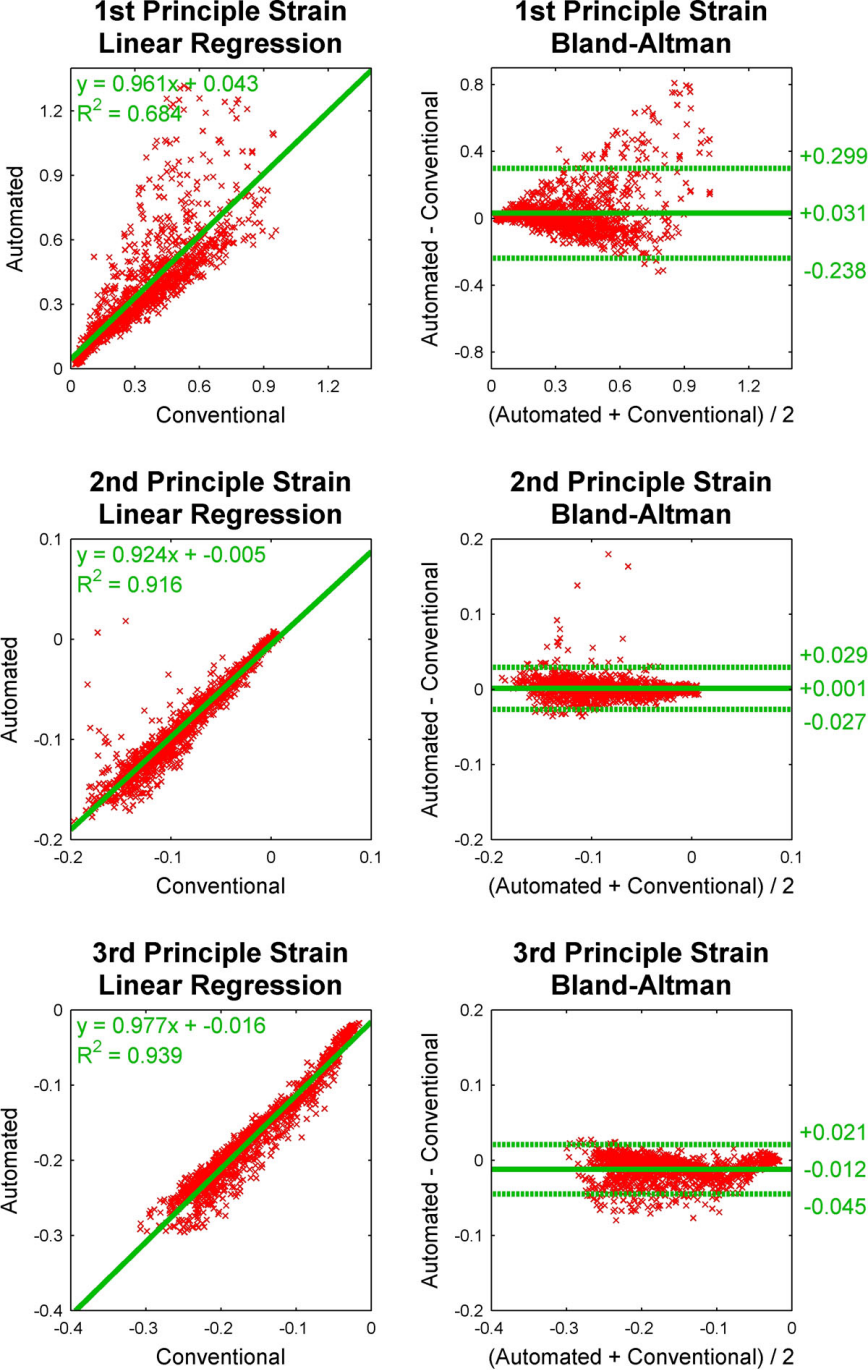
Comparison of regional principle strain values within the 17-segment AHA cardiac model derived from the conventional and automated 3D cine DENSE analyses.

Principle cardiac strains were estimated using the automated algorithm and a conventional method requiring manual anatomical delineation, as illustrated in Fig. 1. The two methods were compared via regional cardiac strain values within a 17-segment AHA model (excluding the apical cap), as illustrated in Fig. 2. For the 1st, 2nd, and 3rd principle strains respectively, linear regression revealed slopes of 0.96 (R^2^=0.68), 0.92 (R^2^=0.92), and 0.98 (R^2^=0.94), and Bland-Altman analyses revealed differences of 0.03±0.27, 0.00±0.03, and <nobr>-0.01±0.03</nobr>.

## Conclusions

This research presented the first fully automated solution to estimate myocardial mechanics from 3D cine DENSE data. Results indicated good agreement between the proposed automated algorithm and a conventional analysis. Ongoing work seeks to further automate analysis by discriminating cardiac anatomy from other tissue.

## Funding

Supported in part by NIH R01 EB 001763 and Siemens Health Care.

